# tsRNA Landscape and Potential Function Network in Subcutaneous and Visceral Pig Adipose Tissue

**DOI:** 10.3390/genes14040782

**Published:** 2023-03-23

**Authors:** Linghui Wang, Hao Gu, Tianci Liao, Yuhang Lei, Yanhao Qiu, Qiuyang Chen, Lei Chen, Shunhua Zhang, Jinyong Wang, Xiaoxia Hao, Dongmei Jiang, Ye Zhao, Lili Niu, Xuewei Li, Linyuan Shen, Mailin Gan, Li Zhu

**Affiliations:** 1 Key Laboratory of Livestock and Poultry Multi-Omics, Ministry of Agriculture and Rural Affairs, College of Animal and Technology, Sichuan Agricultural University, Chengdu 611130, Chinaganmailin@stu.sicau.edu.cn (M.G.); 2 Farm Animal Genetic Resource Exploration and Innovation Key Laboratory of Sichuan Province, Sichuan Agricultural University, Chengdu 611130, China; 3 Chongqing Academy of Animal Science, Chongqing 402460, China

**Keywords:** tsRNAs, adipose tissue, immunity, fatty acid, pig

## Abstract

Noncoding RNAs (ncRNAs) called tsRNAs (tRNA-derived short RNAs) have the ability to regulate gene expression. The information on tsRNAs in fat tissue is, however, limited. By sequencing, identifying, and analyzing tsRNAs using pigs as animal models, this research reports for the first time the characteristics of tsRNAs in subcutaneous adipose tissue (SAT) and visceral adipose tissue (VAT). A total of 474 tsRNAs, 20 and 21 of which were particularly expressed in VAT and SAT, respectively, were found in WAT. According to the analysis of the tsRNA/miRNA/mRNA co-expression network, the tsRNAs with differential expression were primarily engaged in the endocrine and immune systems, which fall under the classification of organic systems, as well as the global and overview maps and lipid metropolis, which fall under the category of metabolism. This research also discovered a connection between the activity of the host tRNA engaged in translation and the production of tsRNAs. This research also discovered that tRF-Gly-GCC-037/tRF-Gly-GCC-042/tRF-Gly-CCC-016 and miR-218a/miR281b may be involved in the regulation of fatty acid metabolism in adipose tissue through SCD based on the tsRNA/miRNA/mRNA/fatty acid network. In conclusion, our findings enrich the understanding of ncRNAs in WAT metabolism and health regulation, as well as reveal the differences between SAT and VAT at the level of tsRNAs.

## 1. Introduction

The worldwide prevalence of obesity nearly tripled between 1975 and 2016. According to the World Health Organization, more than 1.9 billion adults aged 18 and above are overweight, of which more than 650 million are obese (https://www.who.int/, accessed on 11 December 2022). Overweight and obesity have reached a worldwide epidemic level and have become a major challenge in the field of sustainable development worldwide [1]. As a global problem, the risk of obesity is that it can cause many complications, such as diabetes, hypertension, cardiac disease, stroke, and cancer [2,3].

The root cause of obesity and overweight is the energy imbalance between caloric intake and caloric consumption caused by bad eating habits or behavior patterns, which mainly manifests as an abnormal or excessive accumulation of white adipose tissue (WAT) [4]. Therefore, the core of healthy weight control is to control the speed and location of adipose tissue deposition. Adipose tissue remodeling is an important research subject of current health management, and noncoding RNA (ncRNA) is one of the hot research directions [5]. As the most studied ncRNA, the roles of microRNA (miRNA) [6], long noncoding RNA (lncRNA) [7], and circular RNA (circRNA) [8] in regulating adipose tissue development and metabolism have been widely reported. With the development of high-throughput sequencing technology, a class of ncRNAs with a length of about 18–40 nucleotides, which are cut and processed from tRNA and its precursors, has been found in many species, known as tRNA-derived small RNA (tsRNA) [9]. tsRNA is produced by various cleavage events. Therefore, tsRNA can be classified as tRF-5s, tRF-3s, tRF-1s, tRF-2s, and i-tRF according to the position on the precursor or mature tRNA. tRF-5s and tRF-3s are produced from the 5′ and 3′ ends of mature tRNA. tRF-5s is cleaved by the region between the D loop or the D loop and the anticodon loop of the tRNA gene, and it has been reported in mammalian cells, plants, and fission yeast [10,11,12,13,14]. tRF-3s mainly consist of 18 or 22 nucleotides with a CCA at the end [15]. tRF-1s is an RNase Z cleavage product of precursor tRNA produced from the 3′ end of the primary tRNA transcript [10,16]. In addition, there are tRF-2s, which are produced by the cleavage of the anticodon loop of tRNA under hypoxia, and i-tRF, which is mainly derived from the internal regions of mature tRNA (except for the 5′ and 3′ ends). tRFs with a length of 30–36 nt are called tiRNAs (tiRs) or tRNA halves (tRHs). Such tRFs are directly cut from the anticodon loop of mature tRNA and can be divided into 5′ tiRs and 3′ tiRs according to their generated positions [17,18,19]. tsRNA is currently well studied in many organisms and plays a role in many processes. For example, it has been shown that tRFs are able to bind to all four AGO proteins and are involved in the regulation of gene expression [20]. However, tsRNAs are also able to act on gene expression through mechanisms that are not dependent on AGO proteins [21]. In addition, tsRNA plays an important role in regulating the normal growth and development of the body and in the onset and development of disease [22,23].

WAT in the mammalian body can be divided into subcutaneous adipose tissue (SAT) and visceral adipose tissue (VAT). Although adipose tissue generally has the functions of maintaining body temperature, providing energy, immune regulation, buffering impact, etc., it should be noted that adipose tissue in different parts shows great differences in its main functions due to its histological characteristics and adjacent organs [24]. SAT is mainly concentrated below the dermis and above the fascia layer, such as the abdomen, back, and buttocks, and its main function is to maintain body temperature and store energy. VAT is mainly concentrated around abdominal organs, such as the liver, kidney, and gastrointestinal tract, with the function of supporting, stabilizing and protecting internal organs [25]. It has been shown that WNT5A/planar cell polarity (PCP) signaling is hyperactivated in VAT compared to SAT and leads to increased local and systemic inflammation associated with visceral obesity [26]. In addition, in the developmental study of SAT and VAT in Bama pig postnatal development, it was found that genes related to lipid metabolism, REDOX, and other processes were enriched in SAT, while genes related to immunity were preferentially expressed in VAT [27]. In addition, studies have found that SAT and VAT also differ in terms of metabolic components, protein synthesis, transcriptional regulation, etc. For example, lipidomic analysis found that 15 lipidomic metabolites from 104 lipid classes were significantly higher in SAT than in VAT, and the proportion of sphingolipids and glycerophospholipids involved in signaling, endocytosis, and apoptosis was higher in SAT [28]. Proteomic analysis showed that obesity had a greater impact on the protein profile of SAT-derived exosomes than VAT-derived exosomes in DIO mice, and the differentially expressed proteins in SAT and VAT were mainly involved in metabolic processes, such as fatty acid metabolism [29]. In addition, some miRNAs play a role in the pathogenesis of obesity-related cardiovascular and respiratory diseases, and these miRNAs were found to be significantly differentially expressed in SAT and VAT in obese individuals, suggesting that there are differences in transcriptional regulation in SAT and VAT [30]. However, the characteristics and changing patterns of tsRNA expression in SAT and VAT are not clear.

In this study, pigs were used as animal models to identify and analyze the tsRNA of SAT and VAT through high-throughput sequencing, in order to provide a reference for research on the metabolic regulation and remodeling of adipose tissue, as well as new insights for the prevention and treatment of obesity.

## 2. Materials and Methods

### 2.1. Ethics Statement

This research scheme was approved by the Animal Protection and Ethics Committee of Sichuan Agricultural University (Chengdu, China, No. DKY-B20131403). No new experimental animals were slaughtered in this study, and all tissue samples were obtained from our previous study [31].

### 2.2. Animals and Treatment

This study used three pigs (288 day old female Qingyu pig, weight: 107.03 kg) from our previous study [31]. The sacrifice process of pigs referred to good manufacturing practice for livestock and poultry slaughtering—pig (GB/T 19479-2004). After the pigs were slaughtered, the subcutaneous adipose tissue at the back of the last rib and omentum adipose tissue were quickly taken for subsequent experiments. The samples for RNA sequencing were quickly stored in liquid nitrogen, and the samples for tissue sections were fixed in adipose fixation fluid (Servicebio, Wuhan, China) with a volume of 10 times (room temperature). 

### 2.3. tsRNA-seq Data, miRNA Data, and mRNA Data

The tsRNA sequencing dataset was obtained from a previous study [32]. The miRNA data used the same library as tsRNAs, and Bowtie software was used to map clean reads to miRNAs sequences in miRBase (http://www.mirbase.org/, accessed on 18 November 2022). The mRNA data used in this study were from our previous research [33]. We extracted the sequencing data of protein-coding genes (PCG) of deep subcutaneous adipose tissue and greater omental adipose tissue (GSE162145: GSM4943070, GSM4943071, GSM4943072, GSM4943064, GSM4943065, and GSM4943066). 

### 2.4. tsRNA-seq Data Analysis and Target Prediction

The workflow of tsRNAs-seq data processing and analysis was performed as described previously [34]. tRNA sequences of cytoplasmic origin were downloaded from the Genomic tRNA Database (GtRNAdb) [35]. tRNA sequences of mitochondrial origin were predicted with tRNAscan-SE software [36]. Firstly, low-quality reads and adapter sequences were removed to obtain clean reads. Subsequently, Bowtie software was used to map clean data against pig reference genome (Sscrofa11.1) [37]. The EdgeR package was used for differential expression analysis. A |log_2_ fold change| ≥1 and *p* ≤ 0.05 were considered to indicate differentially expressed tsRNAs. The BioMart tool (http://asia.ensembl.org/biomart/martview/49bc7b728830f0d7d598ad482bd71ae2, accessed on 2 November 2022) was used to download the 3′UTR sequences of all genes of pig (Sus scrofa 11.1) for prediction of tsRNA target genes. RNAhybrid (https://bibiserv.cebitec.uni-bielefeld.de/rnahybrid, accessed on 5 November 2022) was used for sequence alignment analysis [33]. Kyoto Gene and Encyclopedia of Genomes (KEGG) pathway enrichment analyses were performed on predicted target genes. 

### 2.5. Tissue Section and Staining

First, 0.5 cm^3^ of SAT and VAT were soaked in adipose tissue fixative solution (G1119, Servicebio, Wuhan, China) and fixed for about 24 h, before dehydration with ethanol ay different concentrations. The tissue was then embedded in paraffin, sliced (8 μm), and finally stained with H&E (hematoxylin and eosin). SAT and VAT sections were observed under a microscope (Nikon, Tokyo, Japan) and photographed.

### 2.6. Analysis of Fatty Acids

Fatty acids were detected using gas chromatography/mass spectrometry (GC–MS 7890B-5977A, Agilent, USA), and the samples were processed as described in our previous experiments [38].

### 2.7. Statistical Analysis

Data wrangling and statistical analysis were performed using WPS Office and SPSS 22.0. Data are presented as the mean ± standard deviation (SD). Differences in phenotypic index and gene expression were determined between two groups using Student’s *t*-test or one-way ANOVA. A *p*-value ≤ 0.05 was considered statistically significant.

## 3. Results

### 3.1. Morphological Characteristics of SAT and VAT in Pigs

**Figure 1:**
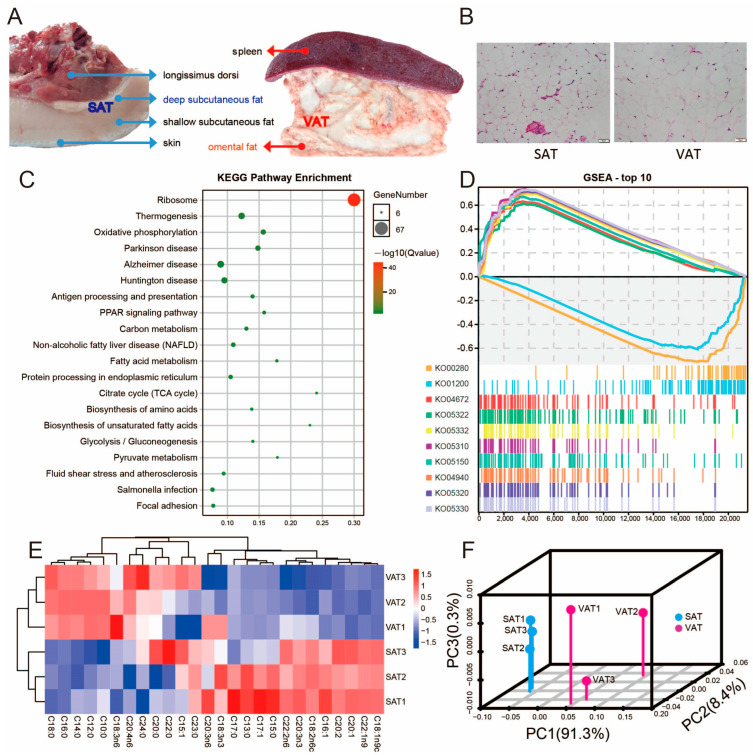
Difference between SAT and VAT. (**A**) Morphological characteristics of subcutaneous adipose tissue (SAT) and visceral adipose tissue (VAT). (**B**) Histological sections of SAT and VAT stained with hematoxylin and eosin (H&E) (200×, 50 μm). (**C**) KEGG (Kyoto Encyclopedia of Genes and Genomes) analysis of the top 500 co-expressed mRNAs in SAT and VAT. (**D**) Gene set enrichment analysis (GSEA) for SAT and VAT. The top 10 (*p*-value) signaling pathways are shown. (**E**) The heat map shows the difference in fatty acid types between SAT and VAT. (**F**) Principal component analysis (PCA) based on differences in fatty acid types (N = 3).

In this study, deep subcutaneous adipose tissue was selected as the representative of SAT, and omental adipose tissue was selected as the representative of VAT. The subcutaneous adipose tissue is adjacent to the longissimus dorsi muscle and skin, divided into deep subcutaneous adipose tissue and shallow subcutaneous adipose tissue (Figure 1A). Omental adipose tissue is adjacent to the spleen and stomach (Figure 1A). The histological sections showed that the adipocytes in SAT were smaller than those in VAT (Figure 1B). The high expression of the top 500 mRNAs in SAT and VAT was mainly related to PPAR, heat generation, fatty acids, and other signal pathways, which is consistent with the main function of adipose tissue (Figure 1C). Gene set enrichment analysis (GSEA) analysis showed that the highly expressed genes in dorsal SAT mainly involved valine, leucine, and isoleucine degradation and carbon metabolism, while the highly expressed genes in VAT were mainly involved in inflammatory diseases, immune diseases, and the immune system (Figure 1D). In terms of fatty acid composition, SAT and VAT also showed different composition rules (Figure 1E,F). 

### 3.2. Characteristics of tsRNA in Pig Adipose Tissue

**Figure 2:**
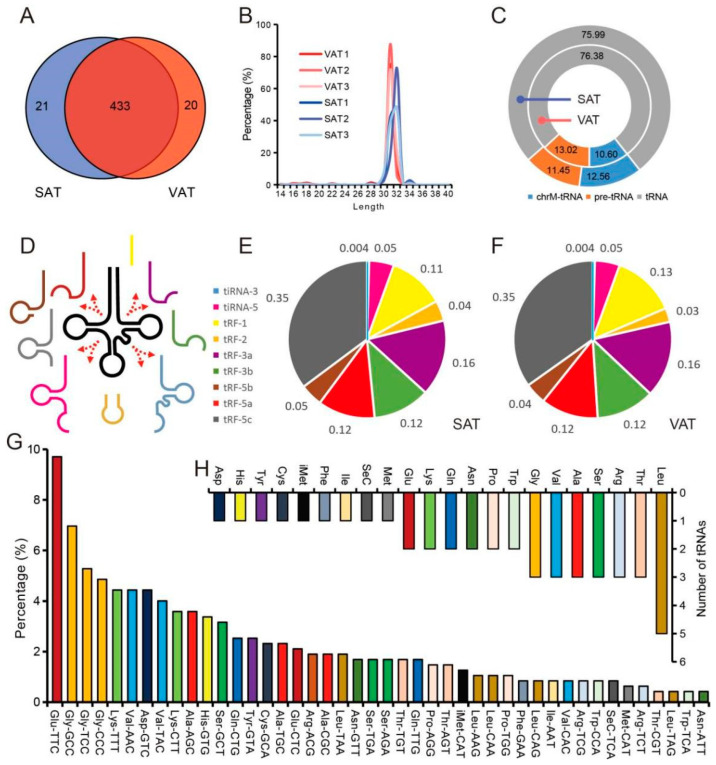
The type and source of tsRNAs in pig adipose tissue. (**A**) Venn diagram showing the number of tsRNAs expressed in SAT and VAT. (**B**) Length characteristics of tsRNAs. (**C**) Source of tsRNAs in pig SAT and VAT (whether from mitochondrial tRNA and whether from tRNA precursor or mature tRNA). (**D**) Schematic diagram of nine types of tsRNAs. (**E**,**F**) Proportion of each type of tsRNA in SAT (**E**) and VAT (**F**). (**G**) Source of tsRNAs in pig SAT and VAT (tRNA). (**H**) Source and amount of tRNA that produced tsRNA.

A total of 474 tsRNAs were identified by high-throughput sequencing in SAT and VAT, which were mainly 28–32 nucleotides in length (Figure 2A,B). According to the source analysis, 11.58% of tsRNAs were derived from mitochondrial tRNAs, and 76.19% of tsRNAs were produced by the cleavage of mature cytosolic tRNAs (Figure 2C). Among the nine kinds of tsRNA, the highest percentage of tRF-5c reads and the lowest percentage of tiRNA-3 reads were determined in both SAT and VAT (Figure 2D–F). On the whole, the tsRNAs of SAT and VAT showed similar patterns in type and source. In addition, statistics showed that these tsRNAs were derived from 44 tRNAs, with the largest number of tsRNAs processed from tRNA-Glu-TTC, accounting for 9.70% (Figure 2G). Further analysis showed that the 44 tRNA isoforms that produce tsRNAs are usually aminoacylated with 22 amino acids, with only one each of tRNA-Asp, tRNA-His, tRNA-Tyr, tRNA-Cys, tRNA-iMet, tRNA-Phe, tRNA-Ile, tRNA-Sec, and tRNA-Met, but five of tRNA-Leu (Figure 2H).

### 3.3. High Expression of tsRNA in Pig Adipose Tissue

**Figure 3:**
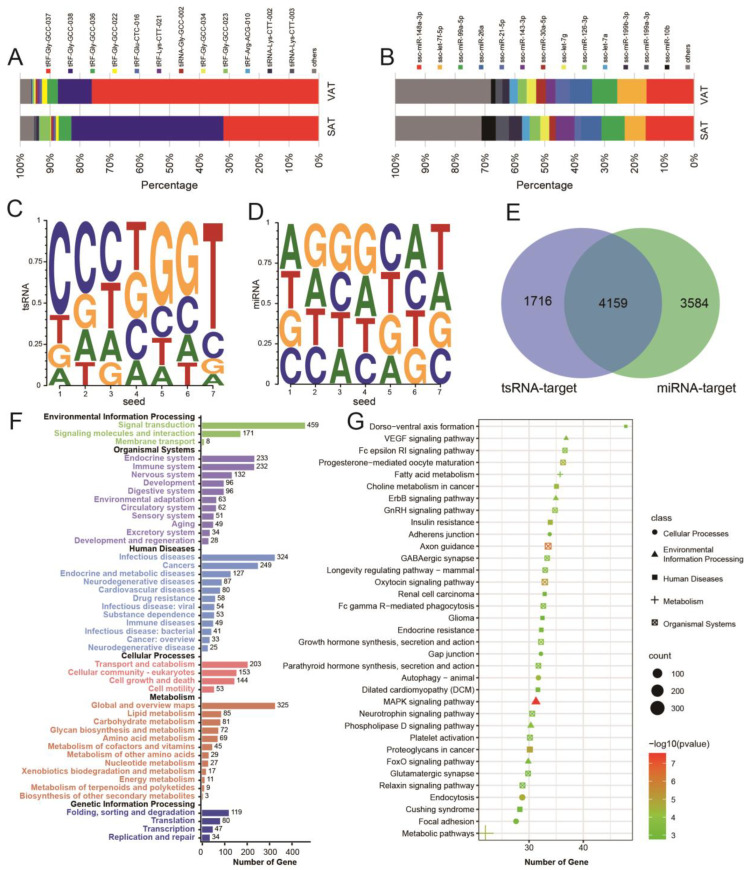
Enrichment analysis of tsRNAs and miRNAs target genes. (**A**,**B**) The proportion of highly expressed tsRNAs (**A**) or miRNAs (**B**). (**C**,**D**) Characteristics of tsRNA (**C**) or miRNA (**D**) Seed Sequences. (**E**) Venn map of target genes of tsRNAs and miRNAs. (**F**,**G**) Enrichment results of KEGG_B_classes (**F**) and pathways (**G**) of co-predicted target genes of tsRNAs and miRNAs.

A total of 433 tsRNAs were co-expressed in SAT and VAT, and 352 miRNAs were co-expressed in SAT and VAT. The top 10 highly expressed tsRNAs accounted for more than 95% of SAT and VAT, while the top 10 highly expressed miRNAs accounted for nearly 70% of miRNAs (Figure 3A,B). Analyzing the seed sequences (2–7 positions) of tsRNAs and miRNAs, it was found that tsRNAs had obvious base preference, while the four bases of miRNAs were evenly distributed (Figure 3C,D). Further study of the target genes of the top 10 highly expressed tsRNAs and the top 10 highly expressed miRNAs revealed that 4159 genes were common target genes of tsRNAs and miRNAs, and these genes accounted for 70.79% of all target genes of tsRNAs and 53.71% of all target genes of miRNAs (Figure 3E). These results indicate that target genes with highly expressed tsRNAs and miRNAs have high coincidence, indicating that their biological functions are similar. Further functional KEGG enrichment analysis of target genes showed that the target genes predicted jointly were mainly enriched in signal transduction, immune system, endocrine system, and other signal pathways (Figure 3F,G). 

### 3.4. Differential Expression of tsRNAs, miRNAs, and mRNAs in Subcutaneous Adipose Tissue and Visceral Adipose Tissue

**Figure 4:**
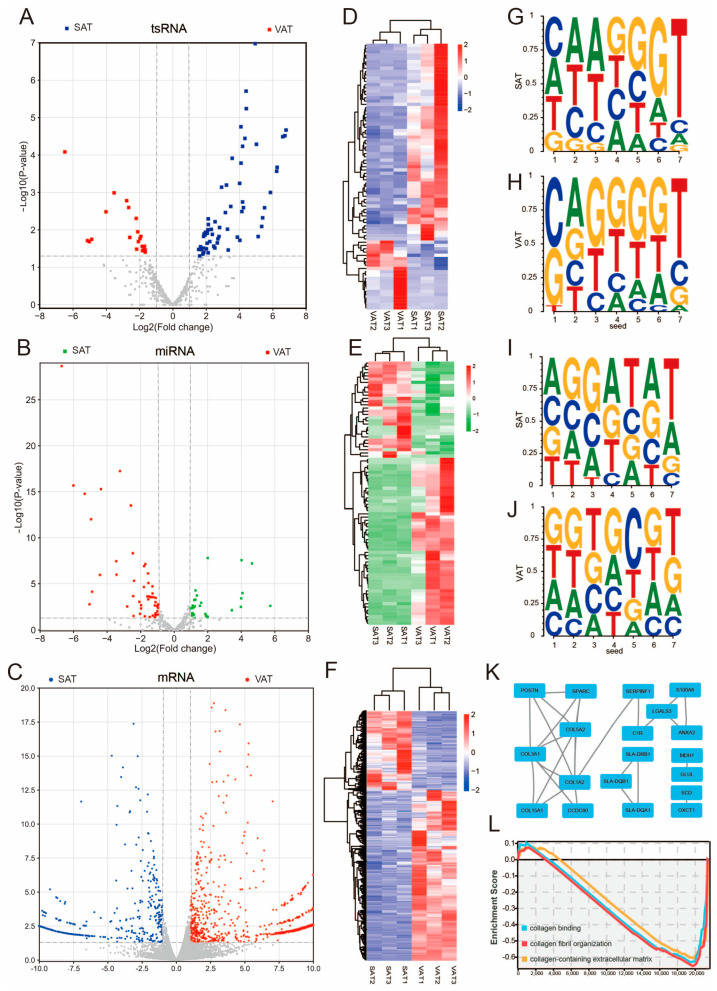
Differential analysis of tsRNAs, miRNAs, and mRNAs in SAT and VAT. (**A**–**C**) Volcano plots showing the tsRNAs (**A**), miRNAs (**B**), and mRNAs (**C**) differentially expressed in SAT and VAT. (**D**–**F**) Cluster analysis of the differentially expressed tsRNAs (**D**), miRNAs (**E**), and mRNAs (**F**). (**G**,**H**) Sequence features of seed sequences that highly express tsRNAs in either SAT (**G**) or VAT (**H**). (**I**,**J**) Sequence features of seed sequences that highly express miRNAs in either SAT (**I**) or VAT (**J**). (**K**) PPI (protein–protein interaction) network analysis of highly expressed and differentially expressed mRNAs in SAT and VAT. (**L**) GSEA of the signaling pathways associated with collagen.

Differential analysis showed that 60 tsRNAs, 30 miRNAs, and 401 mRNAs were highly expressed in SAT, while 21 tsRNAs, 52 miRNAs and 726 mRNAs were highly expressed in VAT (Figure 4A–C). Differentially expressed tsRNAs, miRNAs, and mRNAs were all able to cluster the SAT and VAT tissues into two distinct classes (Figure 4D–F). At the same time, this study also found that the 2–7 high-frequency bases of the highly expressed tsRNAs in SAT and VAT were CAAGGGT and CAGGGGT, respectively (Figure 4G,H). The high-frequency bases at positions 2–7 of the miRNAs differentially expressed in SAT and VAT were also different (Figure 4I,J). The PPI (protein–protein interaction) network analysis of the highly expressed and differentially expressed mRNAs revealed that the core genes were predominantly associated with collagen (COL1A2, COL3A1, COL5A2, COL15A1, and CCDC80) (Figure 4K). GSEA showed that the genes in the signaling pathways associated with collagen were generally highly expressed in SAT (Figure 4L).

### 3.5. tsRNA/miRNA/mRNA Regulatory Network of Subcutaneous Adipose Tissue and Visceral Adipose Tissue

**Figure 5:**
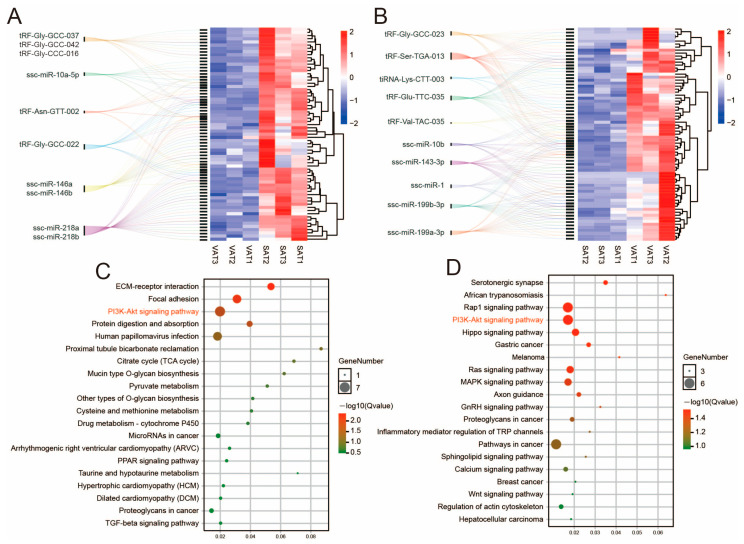
tsRNA/miRNA/mRNA co-expression network. (**A**,**B**) Mulberry leaf plot of the tsRNA/miRNA/mRNA co-expression network based on high mRNA expression in SAT (**A**) or VAT (**B**). (**C**,**D**) KEGG analysis of mRNAs highly expressed in SAT (**C**) or VAT (**D**) in co-expression networks.

We constructed a co-expression network of the tsRNAs/miRNAs/mRNAs in SAT and VAT. The analysis of tsRNA/mRNA and miRNA/mRNA regulatory networks showed that highly expressed mRNAs in SAT were more regulated by miR-218a/miR-218b, while the most highly expressed mRNAs in VAT were regulated by tRF-Ser-TGA-013 (Figure 5A,B). The KEGG analysis of mRNAs in the co-expression network showed that the mRNAs highly expressed in SAT were mainly enriched by the ECM receptor interaction, focal adhesion, and PI3K/Akt signaling pathway (Figure 5C). The mRNAs highly expressed in VAT were mainly enriched by the Hippo signaling pathway, PI3K/Akt signaling pathway, and Rap1 signaling pathway (Figure 5D).

### 3.6. Potential Effects of tsRNA on the Amino Acid Transport Function of Its Host tRNA

**Figure 6:**
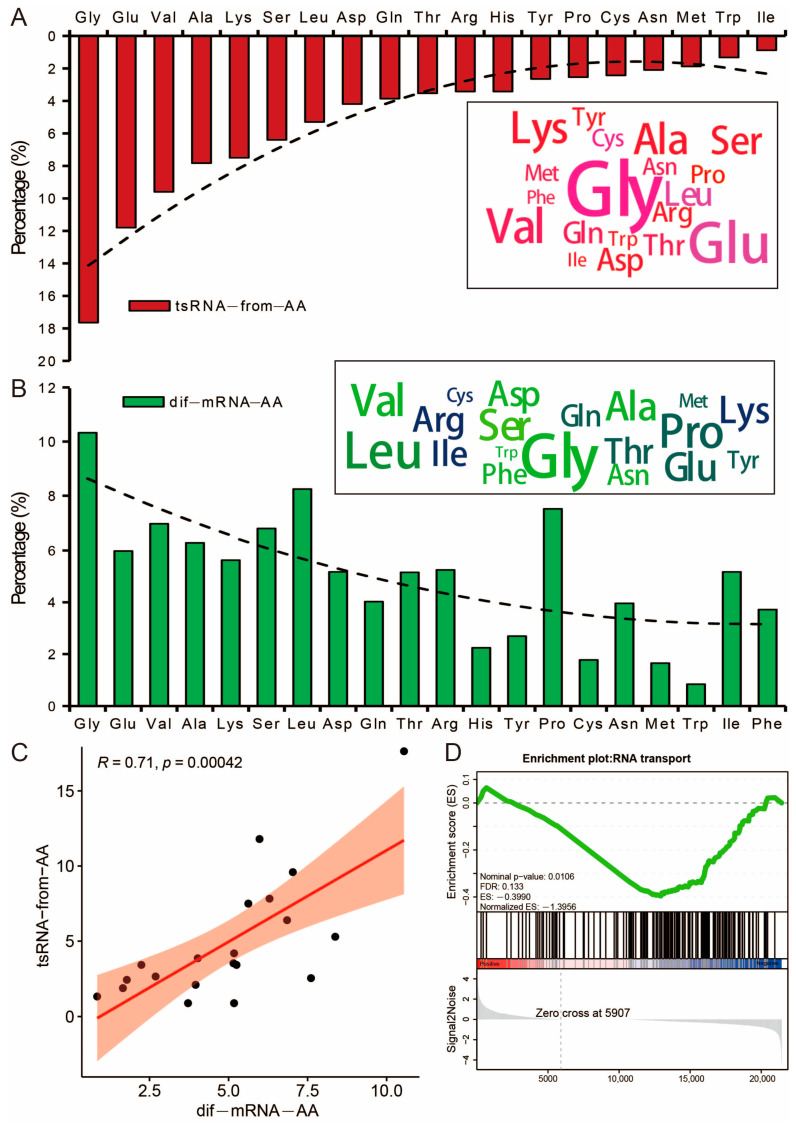
Potential relationship between tsRNA production and host tRNA. (**A**) The portion of tsRNAs based on the classification of transported amino acids. (**B**) Amino acid composition features of the CDS regions of differentially expressed mRNAs. (**C**) Correlation between tsRNA-form-AA and dif-mRNA-AA. (**D**) Results of GSEA of RNA transport.

Further analysis of the parental tRNAs of tsRNAs showed that the number of tsRNAs produced by tRNA-Gly cleavage was the highest percentage of all tsRNAs (17.64%), while the number of tsRNAs produced by tRNA-Ile cleavage was the lowest percentage (0.88%) (Figure 6A). Interestingly, we found that the amino acids corresponding to the codons of differentially expressed genes had a similar pattern. They were highly positively correlated with each other (R = 0.71, *p* = 0.00042) (Figure 6B,C). Further analysis of the RNA transport signal pathway showed that genes related to RNA transport were mainly highly expressed in SAT, and the number of overexpressed tsRNAs in SAT was 2.86 times that in VAT (Figure 4A and Figure 6D). 

### 3.7. Differential tsRNAs and miRNAs Are Involved in the Fatty Acid Metabolism Processes

**Figure 7:**
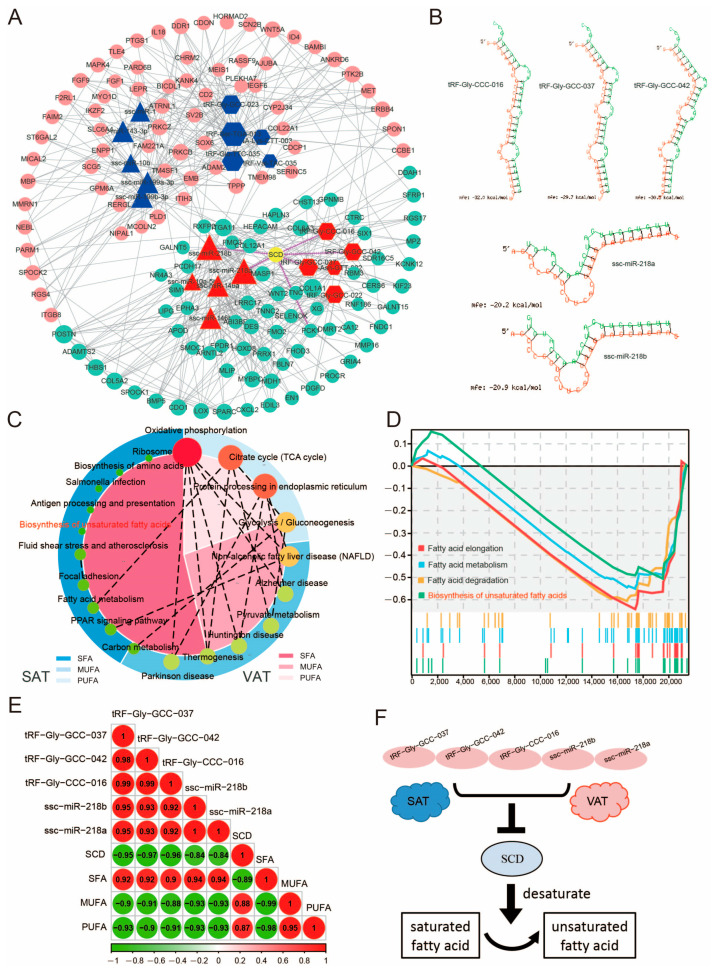
Unsaturated fatty acid metabolism in both the SAT and the VAT. (**A**) Node diagram of the tsRNA/miRNA/mRNA co-expression network. (**B**) Secondary structures of tRF-Gly-GCC-037, tRF-Gly-GCC-042, tRF-Gly-CCC-016, miR-218a, and miR-281b with potential binding sites of the SCD gene (N = 3). (**C**,**D**) Gene set enrichment analysis (GSEA) for SAT and VAT. Fatty acid-related signaling pathways are shown. (**E**) Correlation analysis between genes and different fatty acids. (**F**) Schematic representation of the involvement of tRFs and miRNAs in the control of fatty acid composition in adipose tissue.

Fatty acid metabolism is one of the important basic functions of adipose tissue [39,40]. Analysis of the tsRNA/miRNA/mRNA co-expression network node map revealed that the core gene SCD (stearoyl-CoA desaturase), which is a key fatty acid desaturase [41], may be regulated by both tsRNAs (tRF-Gly-GCC-037, tRF-Gly-GCC-042, and tRF-Gly-CCC-016) and miRNAs (miR-218a and miR-281b) (Figure 4K and Figure 7A). Further analysis found that the free energies of the potential binding sites were all lower than 20 kcal/mol, showing a strong binding relationship (Figure 7B). Analysis of the fatty acid composition of SAT and VAT revealed that the proportion of saturated fatty acid (SFA) present in SAT was significantly lower than that in VAT (Figure 7C). The gene set associated with the biosynthesis of unsaturated fatty acids was also significantly higher in SAT than in VAT (Figure 7D). Further analysis revealed significant negative associations between SCD and tRF-Gly-GCC-037, tRF-Gly-GCC-042, tRF-Gly-CCC-016, miR-218a, and miR-281b, which are involved in the control of fatty acid composition in adipose tissue (Figure 7E,F).

## 4. Discussion

Adipose tissue is regulated by nutrition, as well as nerve and hormone signals, and it widely participates in important biological processes such as temperature maintenance, energy supply, and immune regulation. Moreover, numerous studies have reported that ncRNAs such as miRNAs, lncRNAs, and circRNA in adipose tissue are also involved in the biological function of adipose tissue. However, the biological function of tsRNAs in adipose tissue remains poorly understood. The pig is an ideal animal model for studying adipose tissue function due to its excellent fat deposition ability. Qingyu pigs have stronger fat deposition ability than lean pigs such as Yarkshire pigs; thus, it is more beneficial to study the biological function of adipose tissue. Here, we reported the characteristics of tsRNAs in pig subcutaneous adipose tissue (SAT) and visceral adipose tissue (VAT) by sequencing, as well as predicted and analyzed the potential function of tsRNAs. In addition, we correlated and compared tsRNAs and miRNAs in SAT and VAT due to the similarity in length and function between tsRNAs and miRNAs.

### 4.1. Expression Characteristics of tsRNAs in SAT and VAT

Over 95% of the identified tsRNAs in SAT and VAT were 30–32 nt in length, similar to our previous findings in rat adipose tissue [42]. Since mature tRNAs account for more than 75% of tsRNAs and mature tRNAs are approximately 70–90 nt in length, it can be predicted that the level of tRF-3a, tRF-5a, tRF-2, and tRF-1 in adipose tissue may be relatively low. Consistent with our speculation, analysis of tsRNA types found the highest amount of tRF-5c in adipose tissue, at 35.06%. Several previous studies also found the highest amount of tRF-5c among the identified tsRNAs [43]. This implies that tRNA cleavage in adipose tissue is biased in the production of tRF-5c. Importantly, we also found that the parental tRNAs of tsRNA are highly variable. The number of tsRNAs from tRNA-Glu-TTC accounted for 9.70% of all tsRNAs, while the number of tsRNAs produced by tRNA-Thr-CGT, tRNA-Leu-TAG, tRNA-Trp-TCA, and tRNA-Asn-ATT accounted for only 0.42%. This difference was also reflected in the expression levels of the tsRNAs, with the top 10 highly expressed tsRNAs accounting for more than 95% of the total expression of all tsRNAs, 85.14% of which was contributed by tRF-Gly-GCC-037 and tRF-Gly-GCC-038. Some tsRNAs have miRNA-like functions. For example, it was found that some tsRNAs can bind to AGO proteins in an miRNA-like manner and use seed sequences to bring their target mRNAs into the AGO complex in a similar mechanism to miRNAs [15]. Through sequence feature analysis, we found that the bases at positions 2–7 of tsRNAs had obvious preference, which implied that their potential biological functions also had a tendency, while this characteristic for miRNAs was not obvious. These differences highlight their potential as a tissue type and disease marker.

### 4.2. Potential Function of tsRNAs in Adipose Tissue

The expression levels of genes are usually closely correlated to their function. In the present study, we performed target gene prediction for highly expressed tsRNAs and miRNAs and found that the co-predicted target genes accounted for the majority of all predicted target genes. Functional enrichment analysis showed that highly expressed tsRNAs and miRNAs were mainly enriched in signal transduction, global and overview maps, endocrine system, immune system, and transport and catabolism, which was highly similar to the enrichment results of highly expressed mRNAs, and also coincided with the basic function of adipose tissue [44]. Furthermore, this study also found that the functional enrichment of adipose tissue is closely related to its adjacent tissues. SAT is located in the inner layer of the skin; thus, there are a large number of collagen-associated genes that are highly expressed in SAT. VAT (omental adipose tissue) is closely adjacent to the spleen; thus, the tsRNAs, miRNAs, and mRNAs highly expressed in the VAT are all enriched in a large number of signaling pathways related to inflammation and immunity.

Interestingly, we found that the types of amino acids corresponding to the differential gene codons were highly similar to the types of amino acids transported by the tRNAs that produce tsRNA. Meanwhile, this study found that genes highly expressed in SAT were able to enrich into the RNA transport pathway compared to VAT, and the tsRNAs highly expressed in SAT were also 2.86-fold higher than those in VAT. Fatty acid metabolism is one of the most fundamental biological processes of adipose tissue. Fatty acids can be classified into saturated fatty (SFAs) and unsaturated fatty acids (UFAs) according to whether they contain “C=C” bonds. According to the number of “C=C” bonds, UFAs can be further divided into monounsaturated fatty acids (MUFAs) and polyunsaturated fatty acids (PUFAs) [45]. SFAs are more likely to promote the synthesis of low-density, large volume lipoproteins such as chyle particles, which bring health risks [46]. In this study, we found that the SFA content in SAT was significantly lower than that in VAT; correspondingly, the expression of stearyl CoA desaturase (SCD) in SAT was significantly higher than that in VAT. Similarly, a previous study reported that the SFA content in SAT was lower than VAT, and that SCD1 expression in SAT was higher than VAT in sheep [47]. Interestingly, the tsRNA/miRNA/mRNA co-expression network revealed that SCD may be regulated by both tsRNAs and miRNAs (tRF-Gly-GCC-037, tRF-Gly-GCC-042, tRF-Gly-CCC-016, miR-218a, and miR-281b). A previous study on dairy cows found that miR-218 could be involved in unsaturated fatty acid metabolism through ELOVL5 [48]. ELOVL5 is one of the key enzymes in the synthesis of unsaturated fatty acids, and it has been widely reported to participate in the synthesis of long-chain unsaturated fatty acids in human [49], goat [50], fish [51], and pig [52]. In this study, ELovL5 was also found to be more highly expressed in SAT (fold change = 1.52; *p* = 0.09). These tsRNAs and miRNAs had lower expression in SAT and showed a significant negative correlation with the expression of SCD, which is a candidate marker for important meat quality and production traits in pigs [53]. It should be noted that the expression of three tsRNAs accounted for 76.26% of the total tsRNA expression in VAT, while the miR-218a/miR-218b expression was also ranked in the top 40 positions in the miRNAs of the VAT. Meanwhile, SCD was located in eighth position of highly expressed mRNAs in SAT. These results imply that the tRF-Gly-GCC-037/tRF-Gly-GCC-042/tRF-Gly-CCC-016/miR-218a/miR-281b-SCD-SFA/UFA regulatory network is involved in fatty acid metabolism processes in adipose tissue in different dimensions of tsRNAs, miRNAs, and mRNAs. In the future, we will conduct further research around this regulatory network. The research results on the fatty acid composition of pork also indicate the nutritional value and potential health risks of pig SAT and VAT as human food [54].

## 5. Conclusions

Mammalian white adipose tissue (WAT) can be divided into subcutaneous adipose tissue (SAT) and visceral adipose tissue (VAT) according to its distribution position, which also has differences in physiological functions and metabolic characteristics. In this study, we used pigs to analyze the types and expression characteristics of tsRNAs in SAT and VAT and to construct a preliminary regulatory network for the co-expression of tsRNAs/miRNAs/mRNAs in porcine adipose tissue. The co-expression regulatory network revealed possible molecular evidence (tRF-Gly-GCC-037/tRF-Gly-GCC-042/tRF-Gly-CCC-016/miR-218a/miR-281b-SCD-SFA) that tsRNAs are involved in fatty acid metabolism in adipose tissue, but further studies are needed to confirm this conjecture. Furthermore, we observed a positive correlation between tsRNA production and the degree of tRNA activity. In conclusion, our findings enrich the understanding of ncRNAs in WAT metabolism and health regulation, as well as provide new insights and references for the prevention and treatment of obesity and its related metabolic diseases.

## Data Availability

All data involved in this study can be obtained from the corresponding authors.
